# An overview of the potential sources of diagnostic errors in (classic) thromboelastography curve interpretation and preventive measures

**DOI:** 10.1016/j.plabm.2020.e00193

**Published:** 2020-11-28

**Authors:** Tapasyapreeti Mukhopadhyay, Arulselvi Subramanian

**Affiliations:** a Department of Laboratory Medicine, Jai Prakash Narayan Apex Trauma Centre, All India Institute Medical Sciences, New Delhi, 110029, India; b Room No. 207, Department of Laboratory Medicine, Jai Prakash Narayan Apex Trauma Centre, All India Institute Medical Sciences, New Delhi, 110029, India

## Abstract

Thromboelastography (TEG), a hemostatic point-of-care assay, provides global information about fibrin formation, platelet activation, and clot retraction in real-time. As it is an operator-dependent technique, error in any phase of the testing process can result in the misinterpretation of the thromboelastogram, and subsequently lead to mismanagement of the patient, wastage of blood products besides increasing the financial burden on the hospital and the patient. The present paper describes the possible errors leading to wrong thromboelastogram interpretation, and the respective preventive measure. In the light of limited resources available for operational challenges in TEG, this review paper can prove to be helpful.

## 1 Introduction

Thromboelastography (TEG), invented in 1948, is an assay that detects the contribution of both cellular and plasma components of hemostasis [1]. It provides global information about fibrin formation, platelet activation, and clot retraction in real-time. The ability of TEG to assess the hemostasis in whole blood makes it an ideal point-of-care diagnostic modality for identifying patients with abnormal clot dynamics [2]. It is used bedside or in central laboratories. TEG is widely used in the management of liver transplant patients, in obstetrics, in cardiac surgeries, and for trauma care [3,4]. Multiple studies have found that using a TEG based transfusion algorithm, significantly decreases the number of blood components transfused [[5], [6], [7]].

### 1.1 Working principle of thromboelastography

**Fig. 1:**
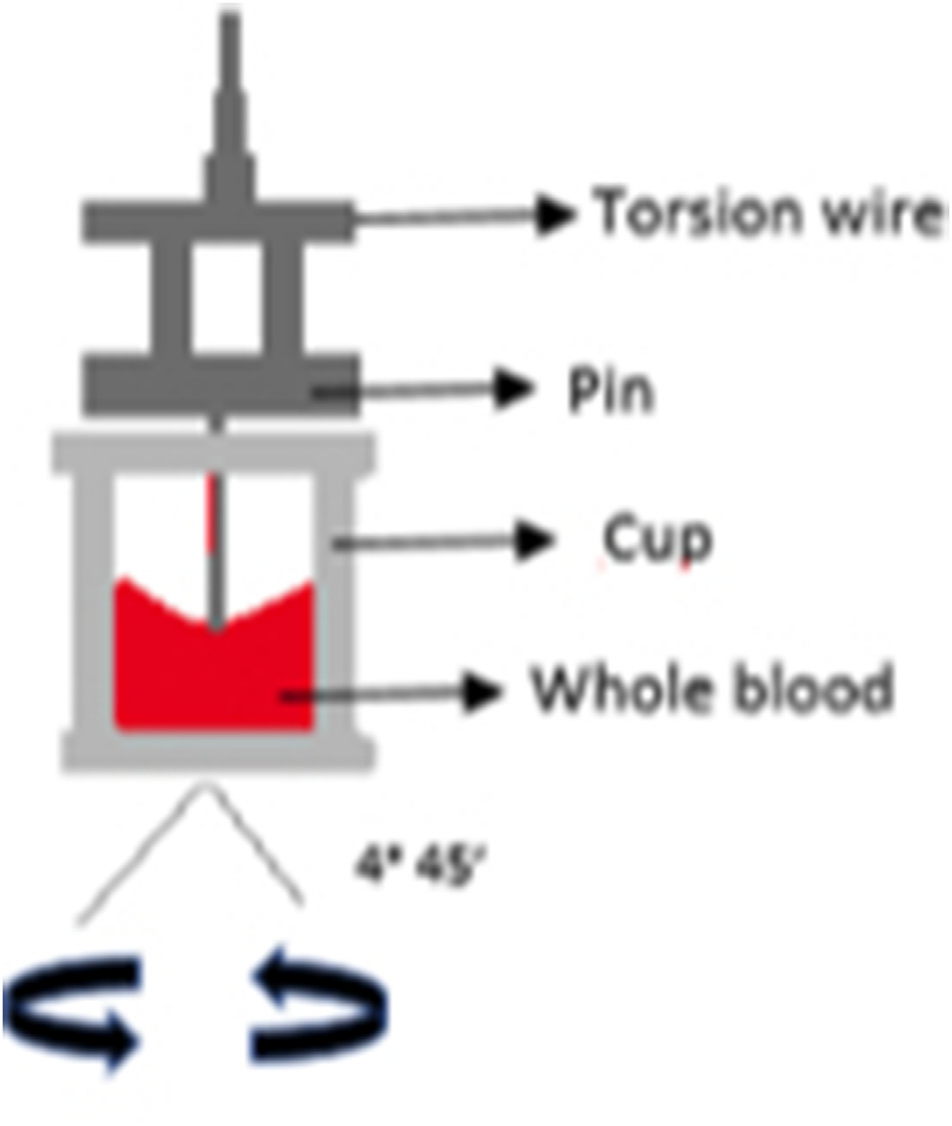
Working principle of Thromboelastography (TEG).

**Fig. 2:**
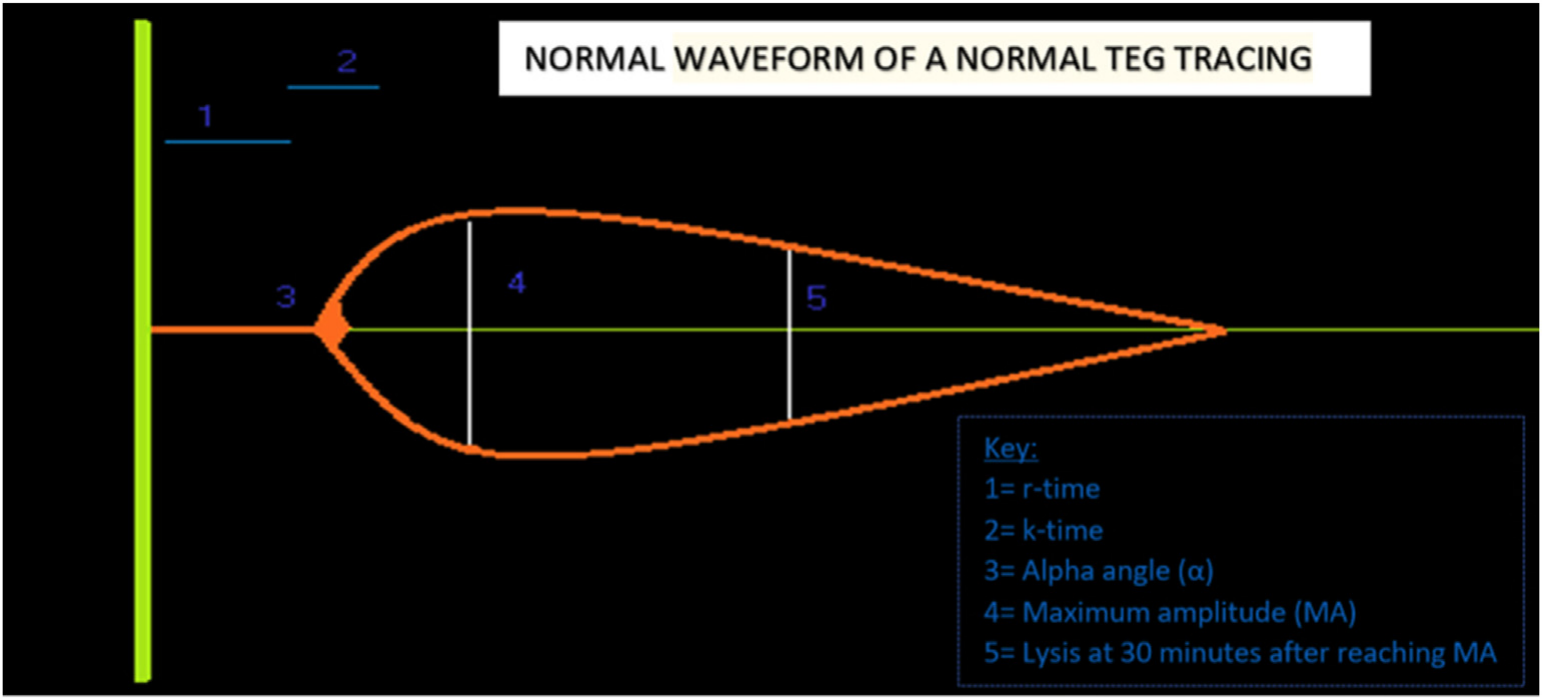
Normal schematic representation of a Thromboelastogram.

Thromboelastography measures the viscoelastic properties of whole blood clot formation under low shear stress. A pin connected to a torsion wire linked to a mechanical-electrical transducer is suspended from above in a cup containing whole blood and calcium chloride, maintained at 37 ​°C as shown in Fig. 1. As the physical properties like the elasticity and strength of the developing clot changes, the rotation of the pin also gets affected and the movement of the pin is converted into electrical signals to create a graphical and a numerical output on the computer. A normal thromboelastogram is schematically represented in Fig. 2. The modern instrumentation can be used to analyze both native whole blood (without any additive) and citrated whole blood by using different initiators [1,8].

The test and the result interpretation are complex, and requires the correlation of medical and treatment history of the patient, and other laboratory parameters like the platelet count, prothrombin time and activated partial thrombolpastin time along with due considerations to the pre-analytical and analytical factors that influence the thromboelastogram. The misinterpretation of the thromboelastogram may lead to mismanagement of the patient, wastage of blood products besides increasing the financial burden on the hospital and the patient [9]. The present paper describes the possible errors leading to wrong thromboelastogram interpretation, based on our 10-year-experience of TEG in a trauma care set up.

### 1.2 Errors in the pre-analytical phase

In general, incorrect laboratory test results are most commonly due to factors occurring outside the laboratory, i.e. in the pre-analytical phase and account for about 70% of all errors [10,11]. These are mostly preventable but are beyond the control of a laboratory personnel. On an average, pre-analytical errors account for 0.23–1.2% of the total hospital expenditure [12]. The common pre-analytical errors affecting a TEG tracing can occur either during sample collection or during transport.

#### 1.2.1 Sources of error during sample collection

Blood samples to be tested could be either run directly after collection without any additive (native blood), often performed at bedside or could be adequately filled in a citrate vial and sent to a centralised laboratory. Although a mislabelled sample is one of the frequent pre-analytical errors but it obviously does not cause any peculiar change in the TEG tracing [13]. Variables that can lead to changes in the TEG tracings are:

##### 1.2.1.1 Use of a wrong vial for blood collection

**Fig. 3:**
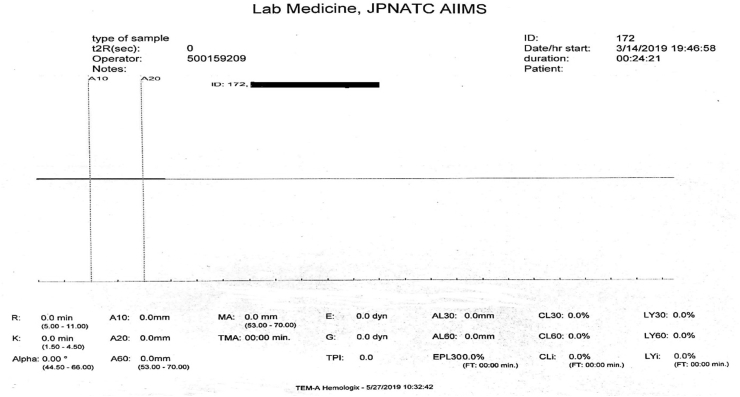
Straight line suggestive of no clot formation.

Use of a vial with a stronger anticoagulant like Ethylenediaminetetraacetic acid (EDTA) produces a straight line (Fig. 3) in TEG even when the patient has normal clot dynamics or is in a hypercoagulable state. This is due to inadequate reversal of calcium chelation in the blood sample by the specified amount of calcium added in vitro.

##### 1.2.1.2 Preventive measure

To collect whole blood up to the ‘mark’ in blue-capped blood collection tube. Nine volume of whole blood is collected in a one volume of 3.2% sodium citrated (0.105–0.109 ​mol/L) ensured by a ‘mark’ on the blue-capped plastic vial [14]. This ratio of 9:1 is critical to ensure minimal osmotic effects and changes in the free calcium concentration in the whole blood [15]. The sample must be inverted several times to mix well after filling the vial to ensure proper mixing. Heparinised blood should be avoided as heparin has enzyme inhibiting property and interferes with end point determination [16]. Sample acceptance and rejection criteria must be defined for the diagnostic test in advance.

##### 1.2.1.3 Incorrect blood withdrawal technique

**Fig. 4:**
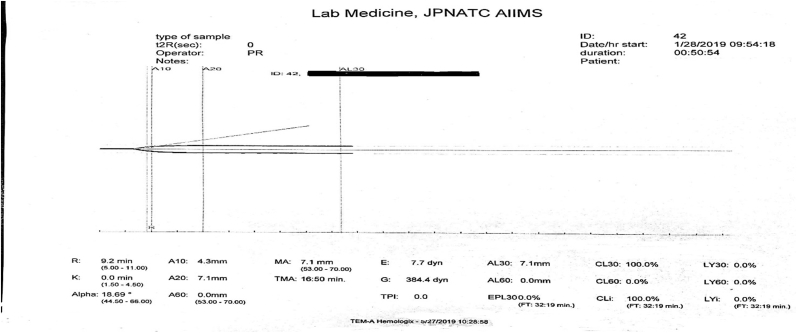
Delayed clot initiation and decreased rate of clot formation with poor clot strength suggestive of a hypocoagulable state.

An already clotted blood sample is invariably rejected. Blood tested from an under filled citrated vial produces a graph representing a hypocoagulable state (Fig. 4) due to the dilution of clotting factors present in the blood with the excess anticoagulant in the vial.

##### 1.2.1.4 Preventive measure

To follow good phlebotomy practice. The importance of the quality of the representative sample received in the laboratory for testing should not be undervalued. The choice of needle size is based on better accessibility for either an arterial or venous sample and should be age appropriate. Blood should be collected keeping in mind the ‘order of draw’ recommended by CSLI and EFLM. [17,18] If blood is drawn too slowly, it may lead to some coagulation before it is tested. Regular training of the phlebotomists and the health care workers regarding the steps of sample collection is recommended.

#### 1.2.2 Sources of error during transport

The two factors that commonly affect a TEG tracing during transport are as follows:

##### 1.2.2.1 Delay in transit

A delay in transport leads to delayed testing and hence loss of clotting factors with shorter plasma half-life [19]. This can give an appearance of false hypocoagulable state on TEG.

##### 1.2.2.2 Preventive measure

**To follow the recommended testing time**. A non-coagulated whole blood has to be run within 4 ​min of collection. It is reliable and preferred where the instrument is placed bed side for immediate results [20]. A citrated sample, is preferred in a set up where the sample is sent to a central laboratory for analysis. Sample is considered stable and useable for up to 2 ​h at room temperature [21]. Any delay may lead to the loss of factors like V, VII and VIII with shorter plasma half-life and may produce a graph representing poor clot dynamics. Time of collection of the sample along with the time of receiving it in the laboratory must be noted. Interpreting a stored sample is not advisable. Although, in a study that used kaolin as an activator, found that the storage of citrated whole blood for up to 30 ​min affected r-time only and not the other parameters [22].

##### 1.2.2.3 Inappropriate handling of blood sample

Excess shaking of the sample during transportation which may cause hemolysis or partial coagulation is unacceptable. In addition, the clotting factors that are mostly proteins, may be lost due to extreme temperature changes during transportation. This may result in absence of clot formation or a hypocoagulable state on TEG (Fig. 3 or Fig. 4 respectively).

##### 1.2.2.4 Preventive measure

To standardise sample handling during transportation. In order to maintain the integrity of the representative sample, shipping the sample in appropriate container and regulate and maintain the optimum temperature during sample transport is preferable [23].

### 1.3 Errors in the analytical phase

The errors occurring in the analytical phase of the testing process are usually the hardest to identify. They may be related to the operator, to the instrument, to the quality of reagents or to the environmental.

#### 1.3.1 Operator related factors

##### 1.3.1.1 Incorrect placement of the cup

**Fig. 5:**
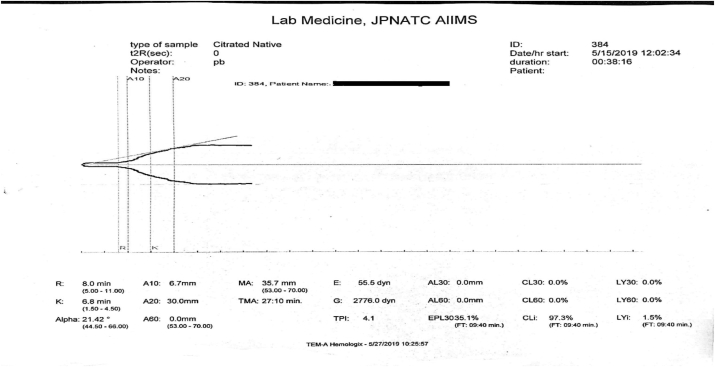
A beak shaped TEG tracing due to incorrect placement of the reaction cup in the instrument socket.

The entire in vitro clot dynamics occur in a reaction cup as described above and thus the end result depends a lot on how the cup has been handled. If the cup is not pushed down precisely by the operator into the specified socket in the TEG machine, then it produces a typical graph with a beak like opening. (Fig. 5). Incorrect placement of the reaction cup results in reduction of the free space of 1 cm between the cup and the pin thus influencing the sheer force that is measured for the formation of the TEG curve.

##### 1.3.1.2 Preventive measure

To appoint a trained personnel dedicated for operating TEG. Like for any other technique or equipment used in the laboratory, one must follow the same stringent quality process for TEG also. A trained personnel, dedicated for operating TEG should be appointed for the daily quality assessment program and maintenance of the instrument [24,25]. Competency assessment of laboratory personnel for operating the machine can be already defined by international bodies can be implemented [26]. To place the reaction cup correctly, gently push the cup down into the machine socket in the cup holder while supporting the cup holder from down. In most machines, final ‘click’ sound denotes that the cup has been placed accurately. Mentioning step-by-step instructions for its start-up, operation and shutdown along with the steps of sample preparation described in manufacturer’s or laboratory’s Standard Operating Procedure (SOP) manuals should be readily available.

##### 1.3.1.3 Incorrect pipetting technique

A blood sample with an inappropriate testing volume or inadequate use of calcium chloride may produce a straight line or a tracing similar to a hypocoagulable state with low values for r-time, k-time and maximum amplitude (Fig. 3 or Fig. 4 respectively).

##### 1.3.1.4 Preventive measure

To use calibrated pipettes by competent operators. Addition of 340 ​μL of citrated blood is re-calcified by the addition of 20 ​μL of 0.2 ​M calcium chloride. Pipetting errors can be reduced by using calibrated pipettes with disposable plastic pipettes or auto diluter pipette tips. It is important to maintain a documented proof of pipette calibration. Also to note, calcium chloride should be pipetted into the reaction cup before pipetting the whole blood. Unnecessary mixing should be avoided to prevent early platelet activation. Auto-pipetting in ROTEM avoids the error [27].

#### 1.3.2 Instrument and accessory related factors

##### 1.3.2.1 Old or expired reagent

Use of old (more than 24 h after reconstitution) or expired calcium chloride solution, or storage at extreme temperatures will hinder the in vitro coagulation cascade taking place in the reaction cup by delaying the initiation of the clotting cascade and result in the production of a hypocoagulable plot (Fig. 4).

##### 1.3.2.2 Preventive measure

To use freshly prepared calcium chloride solution for the test. Calcium has a pivotal role in the coagulation cascade [28]. Citrate being a calcium chelator prevents the blood from clotting before the beginning of the test by binding to the calcium in blood. The use of freshly prepared 0.2 ​M calcium chloride (not older than 24 ​h) stored at room temperature is advised [29].

##### 1.3.2.3 Repeated re-use of the reaction cup

**Fig. 6:**
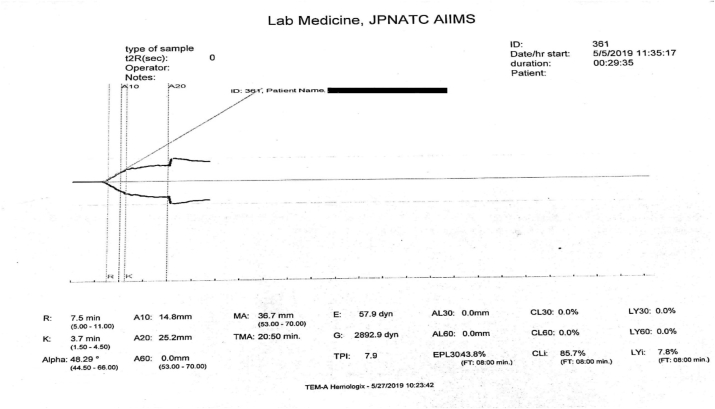
Random jerky tracing due to pin slippage during analysis.

Repeated cleaning of cups with hypochlorite solution or any other cleaning agent leads to shrinking of the internal diameter. Reusing such cups may lead to accidental gripping of the pin during a run. This can lead to a jerk like movement in the pin and thus an abrupt opening of the TEG tracing (Fig. 6).

##### 1.3.2.4 Preventive measure

To use only disposable reaction cups. Cups used to hold the blood sample are made of cyrolite or medical grade polyvinyl chloride [30]. Multiple washing and re-use affects the internal diameter of the well due to deposition of salts and makes it prone to pin gripping. Single use of the disposable cups for each testing process will help reduce errors due to pin gripping.

##### 1.3.2.5 Instrument error due to failed auto-calibration

**Fig. 7:**
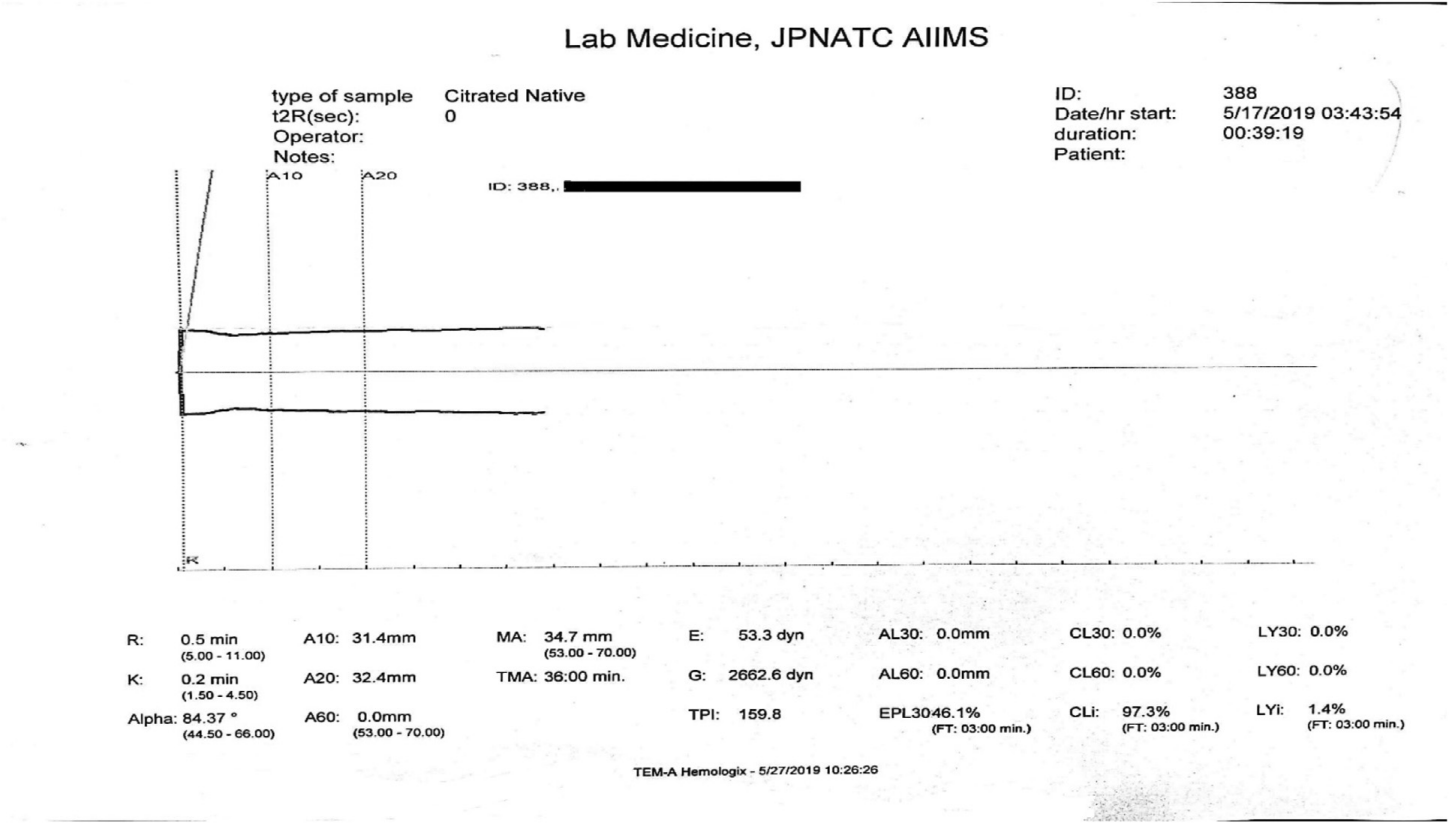
Widely open graph close to the origin appearing to have for increased rate of clot formation and high clot strength suggestive of a hypercoagulable state is actually due to failed auto-calibration before the sample run.

The failure to auto-calibrate before testing a sample leads to the formation of a typical widely open graph starting at the origin (Fig. 7). A very short r-time and k-time, a large alpha angle and a high maximum amplitude is seen in these cases. An inexperienced person may misinterpret it as a hypercoagulable state as it appears to be a case of rapid clot formation.

##### 1.3.2.6 Preventive measure

To perform frequent instrument maintenance. The calibration of an instrument is a critical activity. Calibration and instrument validation is recommended if the instrument has been displaced from one position to the other. Worn out, loose or faulty machinery parts, incorrect procedure and mishandling of the instrument by an untrained operator are common reasons for a failed calibration. Performance checks must be carried out at frequent intervals and recorded. Daily, weekly and monthly maintenance protocols for servicing the instrument should be formulated with the help of the manufacturer’s guideline. The contact number of the company engineer should be displayed and used promptly in case of need. Performance checks and calibration must also be repeated for the repaired equipment after it is returned. The standard 22,870 is applicable to point-of-care testing and is used in conjunction with the standard 15,189 by the International Organization for Standardization (ISO). General guidelines for inspection, measuring and testing laboratory equipment are available in 21 Code of Federal Regulations 820.72 and ISO/IEC 17025:2017 can be referred to [[31], [32], [33]].

##### 1.3.2.7 k-time=0

**Fig. 8:**
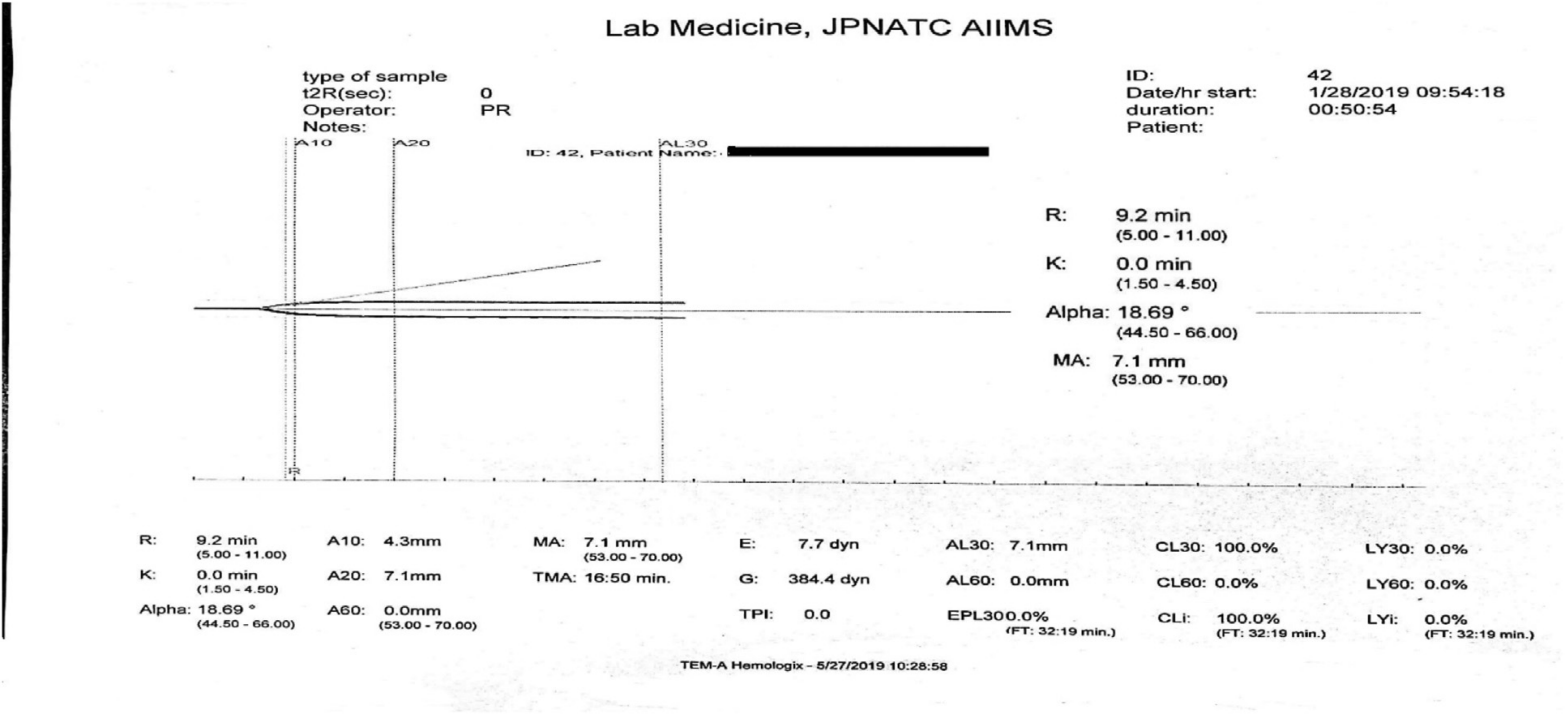
K-time ​= ​0 observed in hypocoagulable state.

A prolonged r-time, zero value of k-time with a low alpha angle and a very low value of maximum amplitude are suggestive of a severe hypocoagulable state. An assessment based only on the value of k-time may reflect a hypercoagulable state instead of a hypocoagulable state (Fig. 8).

##### 1.3.2.8 Preventive measure

To correlate all the TEG parameters visually with the graph. k-time is the time to achieve a certain level of clot strength represented by a point of an amplitude of 20 ​mm [1]. In severe hypocoagulable state, the k-time is not determined correctly as the slope of the graph fails to reach an amplitude of 20 ​mm after the initiation of the clot formation. Thus, correlating clinically and visually assessing the graph with the values of all the TEG parameters simultaneously by a trained laboratory physician will ensure the correct interpretation of the curve.

#### 1.3.3 Environmental factors

##### 1.3.3.1 Disturbance in the surrounding environment

**Fig. 9:**
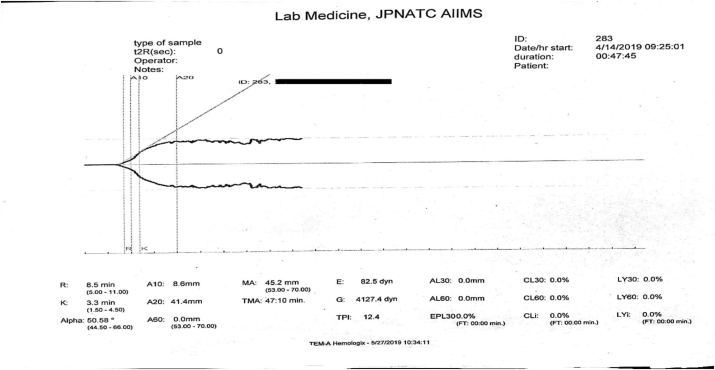
Spikes (continuous or sudden) in TEG tracing are due to environmental disturbances.

Vibrations, also referred to as ‘noise’ which occur at any point during the sample analysis usually reflect as continuous or sudden spikes in the TEG tracing. It is due to the instability of either the pin or the reaction cup or both. It is associated with abnormal values of various amplitudes like amplitude at 10 min (A10) or at 20 min (A20), or maximum amplitude (Fig. 9).

##### 1.3.3.2 Preventive measure

To maintain a vibration-free environment. TEG is a sensitive equipment. Avoiding loud noises, mechanical disturbances or shocks to the instrument and to the platform on which it is placed, are essential to obtain a smooth curve. The displacement of the instrument during or in-between runs should be avoided. The location of equipment in use should be specified in the laboratory equipment inventory. The manufacturer’s guidelines must be followed for transporting the instrument. Workbench space must be sufficient to prevent disturbances [34].

##### 1.3.3.3 Evaporation of the sample from the reaction cup

**Fig. 10:**
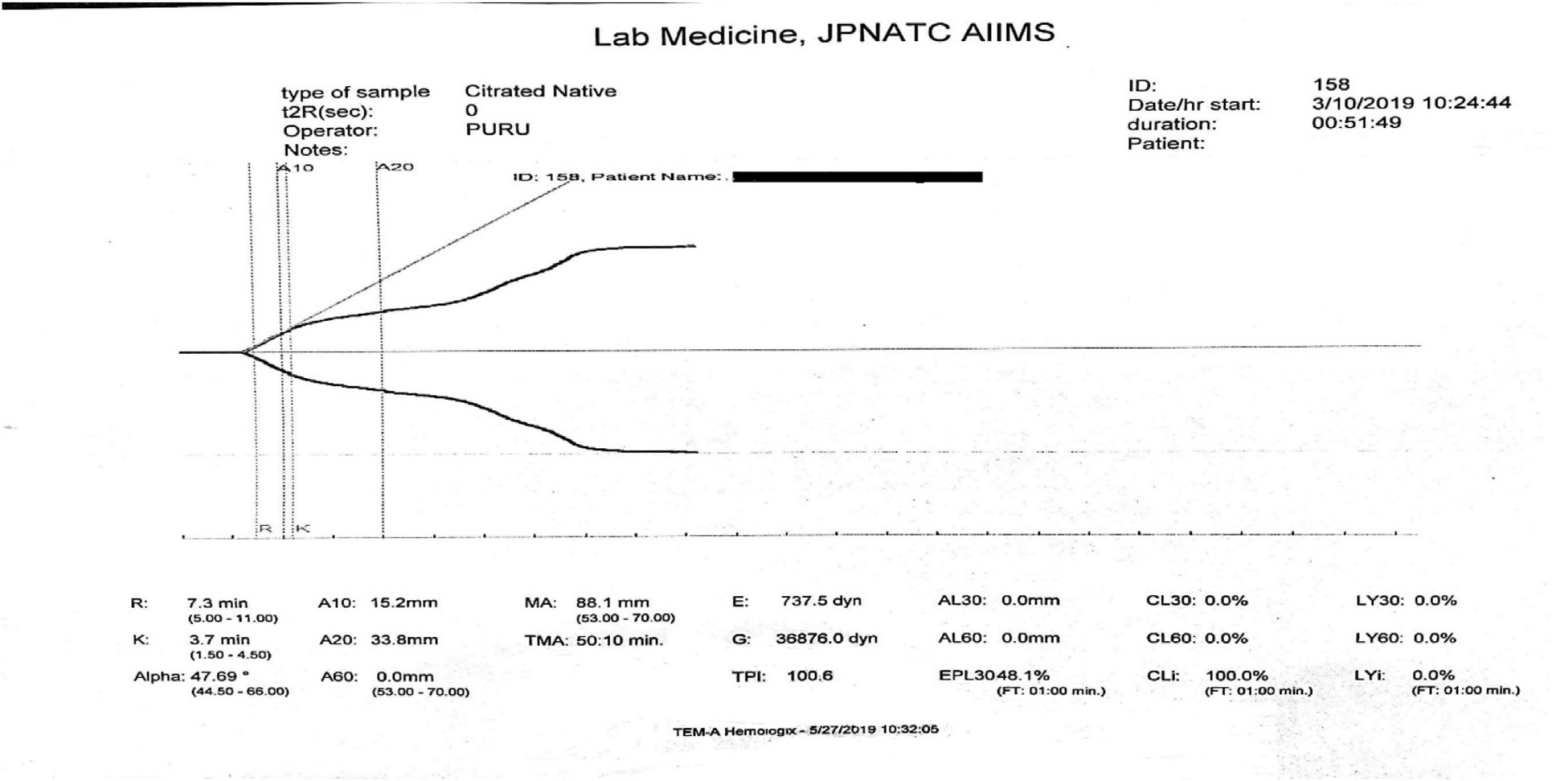
Unpredictable evaporation of sample during the run appears to have very high clot strength suggesting a hypercoagulable state.

Evaporation of the plasma from the sample cup during the sample run alters the plasma to cell (platelets) ratio. The proportion of platelets and its activity become higher than actual, and thus are reflected as higher clot strength or abnormally high value of maximum amplitude in the TEG graph (Fig. 10).

##### 1.3.3.4 Preventive measure

To optimise the humidity and room temperature of the laboratory. Errors due to evaporation of the plasma are generally unpredictable and inevitable. The room and equipment temperatures should be monitored regularly and adjusted whenever required. Depending on the geographical area, a relative humidity of 35–50% and a temperature range of 20–25 ​°C should be maintained for a comfortable working environment [35]. In case it still happens, repeat testing with a fresh sample and allowing it to run up to 30–35 ​min may prevent abnormal results. The laboratory facilities must meet the required environmental conditions, including any needed separation of work areas.

### 1.4 Errors in the post-analytical phase

#### 1.4.1 Delayed test validation and increased turnaround time

Ignored or unidentified errors in the pre-analytical and analytical phases of the total testing process, lead to misinterpretation of test results, delayed validation of test results and increase the turnaround time in the post-analytical phase. The post-analytical errors account for 13–20% of all errors [36]. Even with the advancement of technology, flagging a potentially spurious result is still lacking in the current instrument and thus needs meticulous review of the test results.

#### 1.4.2 Preventive measure

To increase awareness amongst medical professionals. The physician finally interpreting the TEG tracing must be aware and competent to identify the common sources of pre-analytical and analytical errors, and should know the ways to troubleshoot the problems. In addition, the interpretation must be based on the statistically established reference interval for the local population, and it is suggested that each laboratory performing the test should refer to its own list of reference interval [8,37].

The above discussed errors in TEG curve interpretation have been categorised according to the nature of preventability in Table 1. The role of the laboratory physician in the context of emerging technologies is elaborated in an opinion paper [38]. It is the responsibility of the laboratory and the laboratory physician to identify and establish appropriate corrective actions and preventive measures for these errors. Sometimes, it may not be possible to judge the exact error in real-time, especially, if one is not aware of the technical operations involved to obtain the TEG graph. One has to correlate with the patient’s clinical profile and other laboratory parameters while keeping a high index of suspicion for any possible pre-analytical or analytical error. In the light of limited resources available for operational challenges in TEG, this review paper can prove to be helpful.

**Table 1:** Summary of the potential sources of diagnostic errors in TEG interpretation and preventive measures.

Phase of testing process		Sources of error	Preventability	Preventive measures
Pre-analytical phase	During collection	Use of a wrong vial for blood collection	Preventable	To collect whole blood up to the ‘mark’ in blue-capped blood collection tube
		Incorrect blood withdrawal technique	Preventable	To follow good phlebotomy practice
	During transport	Delay in transit	Preventable	To follow the recommended testing time
		Inappropriate sample handling	Preventable	To standardise sample handling during transportation
Analytical phase	Operator related factors	Incorrect placement of the reaction cup	Preventable	To appoint a trained personnel dedicated for operating TEG
		Incorrect pipetting technique	Preventable	To use calibrated pipettes by competent operators
	Instrument and accessory related factors	Old or expired reagent	Preventable	To use freshly prepared calcium chloride solution for the test
		Repeated re-use of the reaction cup	Preventable	To use of only disposable reaction cups
		Instrument error due to failed auto-calibration	Non-preventable but care could have been improved	To perform frequent instrument maintenance
		k–time=0	Non-preventable	To correlate all the TEG parameters visually with the graph
	Environmental factors	Disturbance in the surrounding environment	Preventable	To maintain a vibration-free environment.
		Evaporation of the sample from the reaction cup	Potentially preventable	To optimise the humidity and room temperature of the laboratory.
Post-analytical phase	Delayed validation of test results		Preventable	To increase awareness amongst medical professionals
	Increased the turnaround time		Preventable	

## 2 Conclusion

Strengthening the knowledge of the technical staff and the concerned laboratory physicians on the working principle of thromboelastography, methodological loopholes, and gaining clarity on each step of the testing process will help to anticipate the potential sources of errors and to plan strategies accordingly to overcome them.

## 3 Conflicting interest

Nil.

## CRediT authorship contribution statement

**Tapasyapreeti Mukhopadhyay:** Conceptualization, Funding acquisition, Writing – review & editing, Formal analysis, Concepts, Design, Definition of intellectual content, Literature search, Clinical studies, Experimental studies, Data acquisition, Data analysis, Manuscript preparation, Manuscript editing, Manuscript review. **Arulselvi Subramanian:** Conceptualization, Writing – review & editing, Formal analysis, Concepts, Design, Definition of intellectual content, Literature search, Experimental studies, Data analysis, Manuscript preparation, Manuscript editing, Manuscript review, Guarantor.

## References

[1] Thakur M., Ahmed A.B. (2012). A review of thromboelastography. Gaur A, editor. Int J Perioper Ultrasound Appl Technol.

[2] Perry D.J., Fitzmaurice D.A., Kitchen S., Mackie I.J., Mallett S. (2010). Point-of-care testing in haemostasis: Review. Br. J. Haematol..

[3] Collins S, MacIntyre C, Hewer I. Thromboelastography: Clinical Application, Interpretation, and Transfusion Management. :vol. 7.. 27311154

[4] Trapani L.M. (2013). Thromboelastography: current applications, future directions. Open J. Anesthesiol..

[5] Howley I.W., Haut E.R., Jacobs L., Morrison J.J., Scalea T.M. (2018). Is thromboelastography (TEG)-based resuscitation better than empirical 1:1 transfusion?. Trauma Surg Acute Care Open.

[6] Shore-Lesserson L., Manspeizer H.E., DePerio M., Francis S., Vela-Cantos F., Ergin M.A. (1999). Thromboelastography-guided transfusion algorithm reduces transfusions in complex cardiac surgery. Anesth. Analg..

[7] Enriquez L.J., Shore-Lesserson L. (2009). Point-of-care coagulation testing and transfusion algorithms. Br. J. Anaesth..

[8] Scarpelini S., Rhind S.G., Nascimento B., Tien H., Shek P.N., Peng H.T., Huang H., Pinto R., Speers V., Reis M., Rizoli S.B. (2009). Normal range values for thromboelastography in healthy adult volunteers. Braz. J. Med. Biol. Res..

[9] Laposata M. (2014). Errors in clinical laboratory test selection and result interpretation: commonly unrecognized mistakes as a cause of poor patient outcome. Diagnosis.

[10] Plebani M., Carraro P. (1997). Mistakes in a stat laboratory: types and frequency. Clin. Chem..

[11] Bonini P., Plebani M., Ceriotti F., Rubboli F. (2002). Errors in laboratory medicine. Clin. Chem..

[12] Green S.F. (2013). The cost of poor blood specimen quality and errors in preanalytical processes. Clin. Biochem..

[13] Simundic A.M., Church S., Cornes M.P., Grankvist K., Lippi G., Nybo M., Nikolac N., van Dongen-Lases E., Eker P., Kovalevskaya S., Kristensen G.B., Sprongl L., Sumarac Z. (2015). Compliance of blood sampling procedures with the CLSI H3-A6 guidelines: an observational study by the European Federation of Clinical Chemistry and Laboratory Medicine (EFLM) working group for the preanalytical phase (WG-PRE). Clin. Chem. Lab. Med..

[14] Adcock D.M., Kressin D.C., Marlar R.A. (1997). Effect of 3.2% vs 3.8% sodium citrate concentration on routine coagulation testing. Am. J. Clin. Pathol..

[15] Ingram G.I., Hills M. (1976). The prothrombin time test: effect of varying citrate concentration. Thromb. Haemostasis.

[16] Narayanan S. (2000). The preanalytic phase: an important component of laboratory medicine. Am. J. Clin. Pathol..

[17] Simundic A.M., Bölenius K., Cadamuro J., Church S., Cornes M.P., van Dongen-Lases E.C., Eker P., Erdeljanovic T., Grankvist K., Guimaraes J.T., Hoke R. (2018). Joint EFLM-COLABIOCLI Recommendation for venous blood sampling: v 1.1. June 2018. Clinical Chemistry and Laboratory Medicine (CCLM).

[18] Camenzind V., Bombeli T., Seifert B., Jamnicki M., Popovic D., Pasch T. (2000). Citrate storage affects Thrombelastograph® analysis. Anesthesiology.

[19] Acharya S.S. (2013). Rare bleeding disorders in children: identification and primary care management. Pediatrics.

[20] Shaydakov ME, Blebea J. Thromboelastography (TEG). InStatPearls 2019 Jan 2. StatPearls Publishing. 30725746

[21] Johansson P.I., Bochsen L., Andersen S., Viuff D. (2008). Investigation of the effect of kaolin and tissue factor-activated citrated whole blood, on clot forming variables, as evaluated by thromboelastography. Transfusion (Paris).

[22] NCCLS T. Additives for venous blood specimen collection. Approved Standard—Fifth Edition, H1-A5.;23(33).

[23] Nybo M., Cadamuro J., Cornes M.P., Gómez Rioja R., Grankvist K. (2019). Sample transportation – an overview. Diagnosis.

[24] da Luz L.T., Nascimento B., Rizoli S. (2013). Thrombelastography (TEG®): practical considerations on its clinical use in trauma resuscitation. Scand. J. Trauma Resuscitation Emerg. Med..

[25] MacDonald S.G., Luddington R.J. (2010). Critical factors contributing to the thromboelastography trace. Semin. Thromb. Hemost..

[26] Parikh R.P. (2000). Competency assessment for medical laboratory practitioners and existing rules and regulations. Journal of Health Occupations Education.

[27] Theusinger Oliver Michel, Nürnberg Johannes, Asmis Lars M., Seifert Burkhardt, Rudolf Spahn Donat (2010). Rotation thromboelastometry (ROTEM®) stability and reproducibility over time. Eur. J. Cardio. Thorac. Surg..

[28] Palta S., Saroa R., Palta A. (2014). Overview of the coagulation system. Indian J. Anaesth..

[29] Shida N., Kurasawa R., Maki Y., Toyama Y., Dobashi T., Yamamoto T. (2016). Study of plasma coagulation induced by contact with calcium chloride solution. Soft Matter.

[30] Contreras-García A, Hoemann CD, Wertheimer MR. Thromboelastography Cups and Pins for Improved Blood Coagulation Testing: Surface Modification by Plasma Coating. :1.

[31] Food and Drug Administration Office of Regulatory Affairs. Equipment- ORA-LAB.5.5- FDA.

[32] ISO/IEC 17025 (2017). General Requirements for the Competence of Testing and Calibration Laboratories Section 6.4.

[33] Veselov V., Roytman H., Alquier L. (2012). Medical device regulations for process validation: review of FDA, GHTF, and GAMP requirements. J. Validation Technol..

[34] Facilities and Environmental Conditions - ORA-LAB.5.3 - FDA.

[35] Food And Drug Administration Office Of Regulatory Affairs. ORA Laboratory Manual, Volume III, Section 1 Environmental Health and Safety.

[36] De la Salle B. (2019). Pre-and postanalytical errors in haematology. Int. J. Lit. Humanit..

[37] Subramanian A., Albert V., Saxena R., Agrawal D., Pandey R.M. (2014). Establishing a normal reference range for thromboelastography in North Indian healthy volunteers. Indian J. Pathol. Microbiol..

[38] Orth M., Averina M., Chatzipanagiotou S., Faure G., Haushofer A., Kusec V. (2019). Opinion: redefining the role of the physician in laboratory medicine in the context of emerging technologies, personalised medicine and patient autonomy (‘4P medicine’). J. Clin. Pathol..

